# Protein-Ligand Fishing *in planta* for Biologically Active Natural Products Using Glutathione Transferases

**DOI:** 10.3389/fpls.2018.01659

**Published:** 2018-11-15

**Authors:** David P. Dixon, Robert Edwards

**Affiliations:** ^1^Biophysical Sciences Institute, Durham University, Durham, United Kingdom; ^2^Agriculture, School of Natural and Environmental Sciences, Newcastle University, Newcastle upon Tyne, United Kingdom

**Keywords:** glutathione conjugates, oxylipins, indole derivatives, lignanamides, flavonoids

## Abstract

Screening for natural products which bind to proteins *in planta* has been used to identify ligands of the plant-specific glutathione transferase (GST) tau (U) and phi (F) classes, that are present in large gene families in crops and weeds, but have largely undefined functions. When expressed as recombinant proteins in *Escherichia coli* these proteins have been found to tightly bind a diverse range of natural product ligands, with fatty acid-and porphyrinogen-derivatives associated with GSTUs and a range of heterocyclic compounds with GSTFs. With an interest in detecting the natural binding partners of these proteins *in planta*, we have expressed the two best characterized GSTs from *Arabidopsis thaliana* (*At*), *At*GSTF2 and *At*GSTU19, as *Strep*-tagged fusion proteins *in planta.* Following transient and stable expression in Nicotiana and Arabidopsis, respectively, the GSTs were recovered using Strep-Tactin affinity chromatography and the bound ligands desorbed and characterized by LC-MS. *At*GSTF2 predominantly bound phenolic derivatives including *S*-glutathionylated lignanamides and methylated variants of the flavonols kaempferol and quercetin. *At*GSTU19 captured glutathionylated conjugates of oxylipins, indoles, and lignanamides. Whereas the flavonols and oxylipins appeared to be authentic *in vivo* ligands, the glutathione conjugates of the lignanamides and indoles were artifacts formed during extraction. When tested for their binding characteristics, the previously undescribed indole conjugates were found to be particularly potent inhibitors of *At*GSTU19. Such ligand fishing has the potential to both give new insight into protein function *in planta* as well as identifying novel classes of natural product inhibitors of enzymes of biotechnological interest such as GSTs.

## Introduction

The Arabidopsis genome encodes 55 members of the soluble glutathione transferase (GSTs; EC 2.5.1.18) superfamily, notably members of the plant specific phi (F), tau (U), lambda (L) and dehydroascorbate reductase (DHAR) classes, as well as theta (T), zeta (Z) enzymes (Dixon and Edwards, 2010). The majority of these genes are expressed *in planta* as the respective proteins and show both a complex regulation and sub-cellular localization (Dixon et al., 2009; Jiang et al., 2013; Labrou et al., 2015). As enzymes, plant GSTs can use glutathione (GSH) as either a cofactor, or co-substrate. Activities described to date include the reduction of dehydroascorbate (DHAR), the isomerization of tyrosine degradation products (GSTZ), the reduction of oxidized phenolics (GSTLs) and organic hydroperoxides (GSTF, GSTU, and GSTT) as well as glutathionylating electrophilic natural products and xenobiotics (GSTF and GSTU), including herbicides (Dixon and Edwards, 2010). However, despite displaying this range of enzyme activities *in vitro*, we know relatively little of the function of these proteins *in planta* (Labrou et al., 2015). Using *Arabidopsis thaliana* (*At*) as a model, it has been recognized for some time that the large families of *At*GSTUs and *At*GSTFs (Dixon and Edwards, 2010), are subject to complex regulation in response to infection, abiotic stress, and development (Marrs, 1996; Moons, 2005; Jiang et al., 2013). In some cases, reverse-genetic approaches have confirmed roles for specific GSTs. For example, *At*GSTF12 is involved in the regulation of anthocyanin accumulation (Kitamura et al., 2004), while *At*GSTU20 modulates responses to light reception (Chen et al., 2007) and *At*GSTU17 plays a regulatory role in seedling development (Jiang et al., 2013).

In the case of *At*GSTF12, biochemical function can be linked to the observed phenotype of the knock-out, as inferred through metabolic profiling of the flavonoids present in the respective transparent testa 19 (TT19) mutants (Kitamura et al., 2004; Sun et al., 2012). The *tt19* mutants, which are defective in functional *At*GSTF12 expression, were deficient in anthocyanin and anthocyanidin pigments, while showing elevated levels of flavonol intermediates as compared with wild type plants. Subsequently, *At*GSTF12 was shown to bind the anthocyanins cyanidin and cyanidin-3-*O*-glycoside, but was unable to conjugate them with GSH (Sun et al., 2012). From this it was concluded that *At*GSTF12 is an anthocyanin transporter protein, or ligandin, able to transport these pigments for deposition in the vacuole, through its concerted action with TT12, a tonoplast-localized flavonoid/H+-antiporter (Kitamura et al., 2010). As such, *At*GSTF12 has orthologous functions to other GSTF genes involved in flavonoid accumulation namely, *AN9* in petunia (Mueller et al., 2000), *Fl3* in carnation (Larsen et al., 2003), and *VvGST4* in grapevine (Gomez et al., 2011).

**FIGURE 1:**
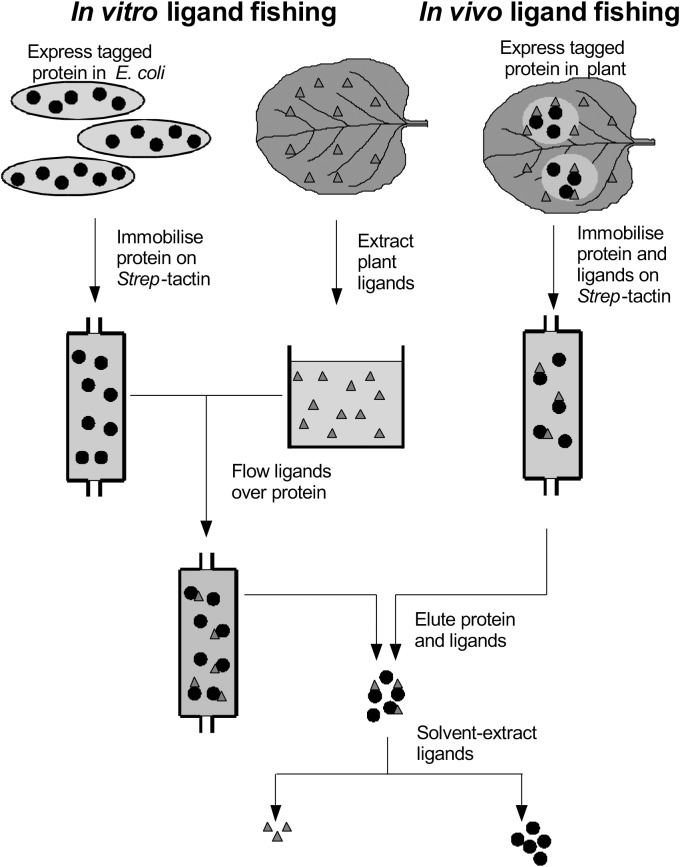
Ligand fishing with Strep-tagged glutathione transferases.

The observation that *At*GSTF12 has a non-enzymic ligand transport, or ligandin function has prompted us to look for binding associations for these proteins in plants, using ‘ligand fishing’ to identify associations with natural products. In its simplest format, individual GSTs are expressed in microbial, or plant hosts, and then subjected to metabolic profiling to identify intermediates that accumulate due to binding interactions with the ‘ligandin.’ Using this methodology with tau class proteins expressed in *Escherichia coli*, we identified that maize (*Zea mays*) *Zm*GSTU1 and *Zm*GSTU2 interacted with porphyrinogen intermediates and caused the hyperaccumulation of colored porphyrins (Dixon et al., 2008). In contrast, when 25 *At*GSTUs were expressed in *E. coli*, they each caused the aberrant accumulation of acylated glutathione thioesters, showing a surprising degree of enzyme-specific ligand selectivity in terms of chain length, oxygenation and desaturation (Dixon and Edwards, 2009). As a refinement to this ligand interaction screening, the GSTs were modified with an N-teminal *Strep*-binding motif, that allowed for the selective recovery of protein-ligand complexes using this tag (Figure 1). The *Strep*-motif was found to be particularly useful in the efficient recovery of fusion proteins, having been developed from a peptide library as the optimal tag-partner of the modified streptavidin termed *Strep*-Tactin (Wisser et al., 2011). Using Strep-tagged *At*GSTUs and *At*GSTFs, we have recovered a variety of ligands from bacteria and plant extracts. These can be broadly divided into a chemically diverse group of natural products that bind as their glutathionylated derivatives (fatty acids, oxylipins, chlorogenic acid), or as a more discreet group of unconjugated natural products derived from indoles, phenols or heterocycles (Dixon et al., 2008; Dixon and Edwards, 2009; Dixon et al., 2011). In the majority of cases, the biological significance of these binding interactions is questionable, as either the ligands available to the tagged GSTs were present in heterologous microbial or plant hosts, or in *in vitro* plant extracts.

In the current study, we now report on a directed and systematic search for natural products that selectively bind to the two best characterized and abundant GSTs in Arabidopsis, namely *At*GSTU19 and *At*GSTF2, when these proteins are expressed in living plants. The strategy adopted has used the *Strep*-tagged GSTs as protein ‘hooks,’ which when either expressed *in planta* transiently (*Nicotiana benthamiana*), or stably (Arabidopsis) bind endogenous ligands in the host which can then be recovered following affinity capture of the fusion protein (Figure 1). Any compounds recovered have then been characterized in detail by high resolution mass spectrometry (MS) and by reference to prepared standards.

## Materials and Methods

### Preparation of Reference Ligands

Kaempferol and kaempferol-3,4′-dimethyl were obtained from Apin (Abingdon, United Kingdom). Other flavonols were extracted from the surface of *N. benthamiana* by dipping 200 g of intact leaves in 500 ml methanol. The extract was concentrated, clarified by centrifugation and hexane added to 20% v/v. After centrifugation, the aqueous phase was applied onto an Accubond II C18 SPE column (1000 mg; Agilent Technologies United Kingdom Ltd., Stockport, United Kingdom). After washing with methanol:water (1:1), flavonols were recovered in methanol (5 ml) and individually purified by preparative reversed phase HPLC as described (Dixon et al., 2012), but using a 5–100% gradient of acetonitrile. Flavonols were quantified by UV absorbance, assuming ε_351__nm_ = 14 mM^-1^.

To generate 3-methylindolyl glutathionyl disulfide (ISSG), *bis*-(3-indolemethyl) disulfide (ISSI) was synthesized as described (Himel et al., 1962) and reduced to 3-thiomethylindole (ISH) by mixing 125 μl of 165 mM ISSI in dimethyl sulphoxide (DMSO) with 125 μl of 200 mM tris-(2-carboxyethyl)phosphine (TCEP) in DMSO and 125 μl of 1.0 M Tris-Cl pH 7.5. After 30 min at 22°C, 750 μl of 0.1M GSH pH 7.0 was added together with 375 μl DMSO and 40 μl of 30% H_2_O_2_, and the reaction incubated for 16 h at 22°C. ISSG was purified from the reaction mixture by preparative reversed phase HPLC, and quantified by UV absorbance, assuming ε_280__nm_ = 5 mM^-1^. N-*trans*-feruloyltyramine was prepared as described (Villegas and Brodelius, 1990). Mixed lignanamides were formed by reacting 40 mM feruloyltyramine in methanol at 20°C for 1 h with one volume of 2.5% w/v aqueous solution of FeCl_3_ (Sakakibara et al., 1992). Products were extracted with ethyl acetate, partitioned against water and the organic phase dried and re-dissolved in methanol. Lignanamide-GSH conjugates were synthesized by incubating 160 μM feruloyltyramine (or mixed lignanamides) for 30 min at 20°C with 0.1 μM horseradish peroxidase, 1 mM GSH and 200 μM H_2_O_2_ in 50 mM Tris-Cl pH 7.5.

### Enzyme and Ligand Binding Assays

Isothermal titration calorimetry (ITC) experiments were performed at 25°C in HBS buffer and analyzed using a VP-ITC instrument with Origin 7.0 software (GE Healthcare, Amersham, United Kingdom). Where ligands were added in solvents, an equivalent volume of ethanol was used as control. With the flavonols, 0.1% w/v Tween 20 was used to increase ligand solubility. In addition to titrating ligand into protein, control titrations of ligand into buffer and buffer into protein were performed. For studies with *At*GSTF2, the respective Strep-tagged protein was purified from *E. coli* grown in minimal medium to minimize contamination with pre-bound ligands and used in ITC assuming 1:1 binding to ligand, as previously determined (Dixon et al., 2011).

### Plant Studies

Untransformed Arabidopsis root cultures were grown as described (DeRidder et al., 2002), without illumination. GST coding sequences were sub-cloned into BIN-STRP3 to allow constitutive and transient expression of N-terminally *Strep*-tagged enzymes in Arabidopsis and Nicotiana, respectively (Dixon et al., 2009). Stable transformants of arabidopsis (ecotype Columbia) were generated by floral dipping (Clough and Bent, 1998). Control plants were generated by transforming with the empty vector pCAMBIA3300^1^. Transformants were selected by spraying soil-grown seedlings with 0.02% w/v glufosinate ammonium, and lines expressing high levels of recombinant protein chosen by western blotting using alkaline phosphatase-linked *Strep*-Tactin as probe. Herbicide-resistant T2 and T3 plants were used for pull-down experiments. GSTs were purified from frozen plant tissue using 4 v/w 100 mM Tris-Cl pH 7.5 containing 150 mM NaCl, 1 mM EDTA, 50 μg/ml avidin, 10 μg.ml^-1^ bovine pancreatic DNase I, 10 μg.ml^-1^ bovine pancreatic RNase A and 5% w/v insoluble polyvinylpolypyrrolidone. For *N. benthamiana* extractions, 10 mM sodium ascorbate was also included. After filtration through miracloth (Calbiochem) and clarification by centrifugation (15,000 *g*, 20 min, 4°C), the *Strep*-GSTs were affinity purified as described (Dixon and Edwards, 2009). Purified proteins were concentrated to approx. 100 μl by ultrafiltration through a 10 kDa-cutoff membrane (2 ml Vivaspin; Sartorius Stedim United Kingdom Ltd., Epsom, United Kingdom). Ligands from bacterial, plant and *in vitro* pull-downs were analyzed by HPLC-MS as described (Dixon and Edwards, 2009).

## Results

### Fishing for Ligands of Plant GSTs *in planta*: Transient Expression in *N. benthamiana*

**FIGURE 2:**
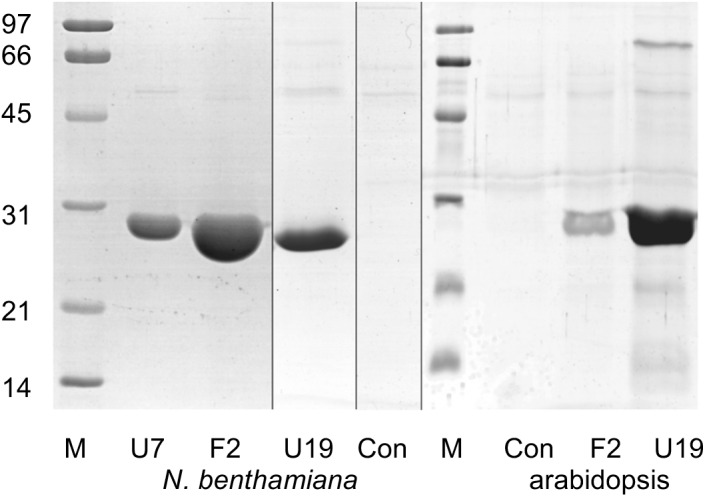
Composite image showing Coomassie Brilliant Blue-stained SDS-PAGE analysis of GST purified by *Strep*-tactin affinity chromatography after expression in *Nicotiana benthamiana* and arabidopsis. Lane M, molecular weight markers (sizes as indicated, in kDa); U7, *At*GSTU7; F2, *At*GSTF2; U19, *At*GSTU19; Con, infiltration control.

**FIGURE 3:**
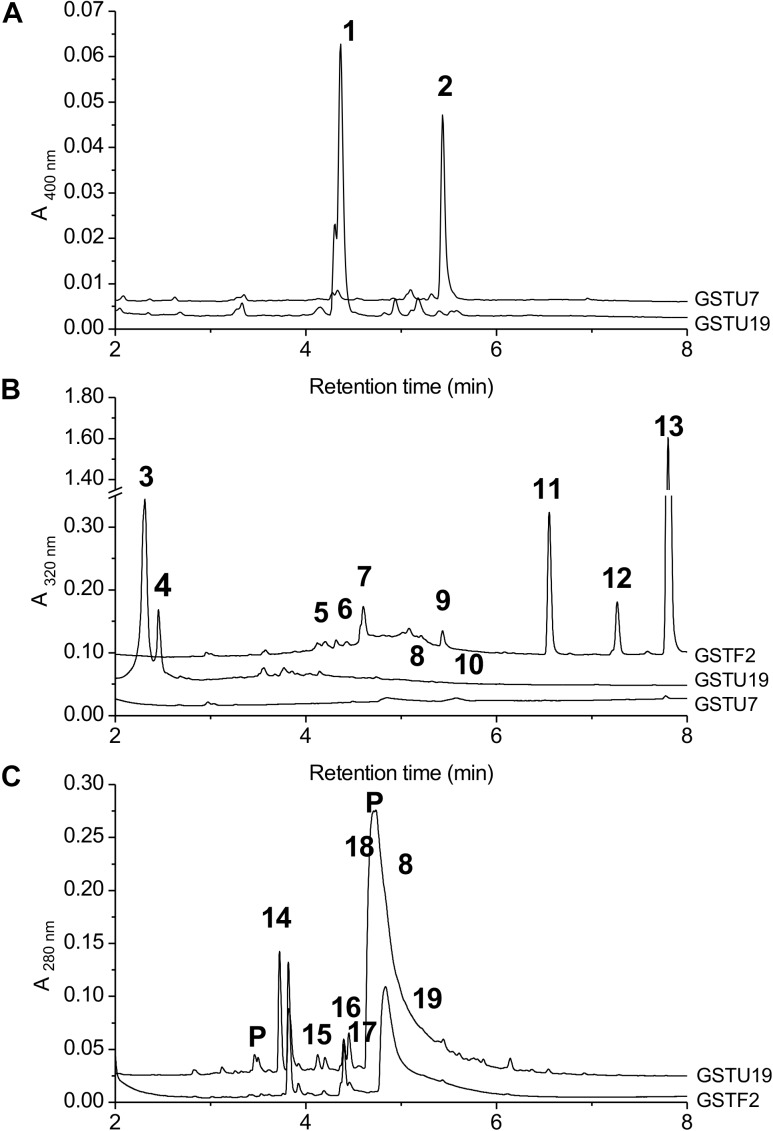
HPLC-MS of GST ligands described in the text. **(A)** Extracts from bacteria expressing GSTU19 (black line) or GSTU7 (gray line), showing accumulation of porphyrin metabolites **1** and **2**. No major peaks at 400 nm were observed in comparable extracts from control bacteria. **(B)** Bound ligands recovered from purified *Strep*-tagged GSTU7, GSTU19 and GSTF2 transiently expressed in *N. benthamiana*, showing additional ligands **5**–**13** bound to GSTF2 and ligands **3**, **4**, **8,** and **10** bound to GSTU19. **(C)** Bound ligands from purified GSTF2 (gray line) and GSTU19 (black line) following stable expression in arabidopsis showing additional ligands **8** and **14**–**19** bound to GSTU19. Peaks labeled “P” were GSTs or proteolysis-derived oligopeptide fragments.

**FIGURE 4:**
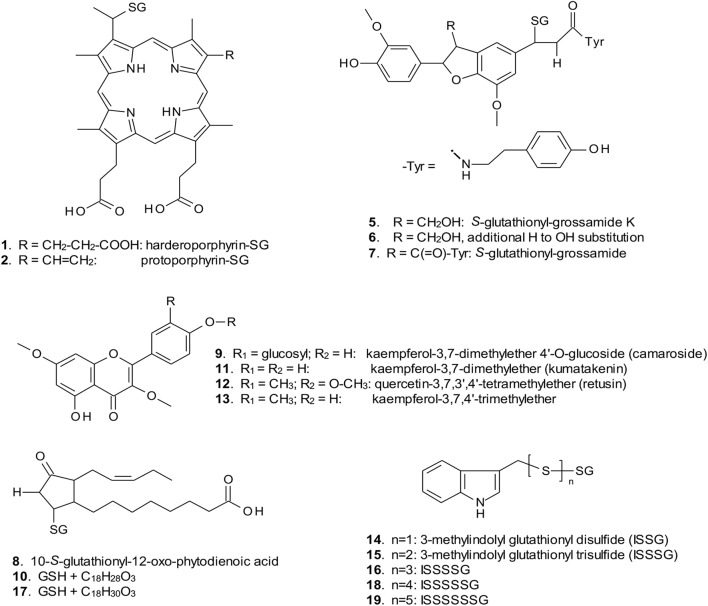
Structures of GST ligands isolated from bacteria and plants in current and earlier studies. SG, *S*-glutathionyl. For ligands **2**, **5**–**7**, **9**, **11**, **12**, and **13**, multiple possible structural isomers exist, and one example is shown.

*At*GSTF2 and *At*GSTU19 were individually transformed into the leaves of *N. benthamiana* through *Agrobacterium* infection using vacuum infiltration. In addition, *At*GSTU7, a tau class enzyme showing similar binding activities to *At*GSTU19 (Dixon and Edwards, 2009), was used in early studies for comparative purposes. After 3 days, the infiltrated leaves (50 g) were harvested and the *Strep*-tagged proteins purified from crude extracts using *Strep*-Tactin affinity chromatography. Both *Strep*-tagged GSTs were strongly expressed in *N. benthamiana* and were recovered in good yields as the pure proteins (Figure 2). Having immobilized the GSTs on the *Strep*-Tactin columns, the washed proteins were selectively desorbed using desthiobiotin and then solvent-extracted with methanol. The bound ligands were then analyzed by HPLC-MS (Figure 3), with the chemical characteristics of each metabolite identified summarized in Table 1. For reference, the ligands recovered when the Strp-tagged proteins were expressed in *E. coli* is shown (Figure 1). In each case, the deduced identities of the ligands are summarized in Figure 4, with each ligand assigned a unique number (**1–19**).

**Table 1:** Properties of GST ligands identified by HPLC-MS.

Peak	λ_*max*_ (nm)	*m/z*	Likely identity
1	394	916.374	harderoporphyrin-SG
2	399	870.356	protoporphyrin-SG
3	252, 323	660.163	chlorogenic acid-SG
4	252, 314	660.165	isomer of chlorogenic acid-SG
5	277	799.276	grossamide K-SG
6	284	815.270	hydroxy-grossamide K-SG
7	283, 307 (sh)	932.326	grossamide-SG, cannabisin D-SG
8	–	600.283	10-*S*-glutathionyl-12-oxo-phytodienoic acid
9	265, 344	477.127	kaempferol-3,7′-dimethylether-4′-*O*-glucoside
10	–	600.284	oxylipin-SG
11	267, 348	315.080	kaempferol-3,7′-dimethylether
12	253, 268 (sh), 353	359.105	quercetin-3,7,3′,4′-tetramethylether
13	267, 346	329.103	kaempferol-3,7,4′-trimethylether
14	279	469.121	ISSG
15	272, 374	501.094	ISSSG
16	272, 375	533.068	ISSSSG
17	–	602.312	oxylipin-SG
18	Not resolved	565.037	ISSSSSG
19	Not resolved	597.001	ISSSSSSG

*Compounds **1** and **2** bound *At*GSTU19 and *At*GSTU7, respectively in *Escherichia coli*, compounds **3**, **4**, **8**, and **10** bound *At*GSTU19 in *Nicotiana benthamiana* (Dixon and Edwards, 2009), compounds **5**–**13** bound GSTF2 in *N. benthamiana* and compounds **8** and **14**–**19** bound *At*GSTU19 in arabidopsis. Sh, shoulder; SG, *S*-glutathione; IS, 3-thiomethylindole. Compound structures are illustrated in Figure 2 and CID mass and UV spectra are as published (Dixon and Edwards, 2009), or are shown in Supplementary Figure S1*.

When over-expressed in *E. coli* grown on minimal media as described previously (Dixon et al., 2008), the recovered Strep-tagged GSTU proteins were found to exclusively retain glutathionylated porphyrins (Figure 3A). While *At*GSTU7 preferentially retained the protoporphyrin conjugate (**2**), *At*GSTU19 showed a marked preference for the harderoporphyrin (**1**) derivative. These metabolites were not retained by *At*GSTF2, neither were they identified in any of the subsequent plant expression studies. When expressed in Nicotiana, *At*GSTU19 was found to bind multiple metabolites (Figure 3B), which on the basis of their mass spectra (Dixon and Edwards, 2009), could be identified as glutathionylated conjugates of chlorogenic acid (**3**, **4**) and oxylipins (**8**, **10**). The other tau protein, *At*GSTU7 was not found to bind any unique ligands and was not analyzed further. *At*GSTF2 bound similar oxylipin conjugates, as well as retaining metabolites which based on their UV absorbance spectra, were aromatic in nature (compounds **5** to **13**; Figure 3B). The more polar ligands (compounds **5**, **6,** and **7),** had the characteristic UV spectra of phenolics. In each case, tandem MS analysis showed neutral losses of fragments of 75 Da, 129 Da, and 307 Da, along with an *m/z* 308 ion, characteristic of glutathione conjugates (Supplementary Figure S1). Compound **7** was the most abundant of these ligands, with its properties consistent with it being a glutathione conjugate of the condensation product of two *trans-N*-feruloyltyramine molecules, such as grossamide (Yoshihara et al., 1981), or cannabisin D (Sakakibara et al., 1992). Previously undescribed in the literature, such a conjugate would be most likely formed from the 1,4-Michael addition of GSH to the α, β-unsaturated amide (Figure 4). By analogy, **5** was most likely a glutathionylated derivative of grossamide K (Table 1 and Figure 4), which consists of feruloyltyramine condensed with coniferyl alcohol (Seca et al., 2001). Compound **6** appeared to be a glutathionylated derivative of the condensate of feruloyltyramine with hydroxyconiferyl alcohol (Table 1 and Figure 4). By way of confirmation of these structural assignments, orthologues of **7** were synthesized by the oxidative glutathionylation of feruloyltyramine. The resulting products were shown to have near identical chromatographic characteristics and MS spectra to **7**, consistent with them being structural isomers of the *At*GSTF2 ligand (Supplementary Figure S2). To investigate the origins of the conjugated ligand, *At*GSTU19 was incubated with feruloyltyramine and GSH. *At*GSTU19 was unable to catalyze the formation of the conjugate indicating that the ligands had been generated by an alternative mechanism.

The UV spectra of the more hydrophobic compounds **9**, **11**, **12,** and **13** were consistent with them being related to flavonoids. To characterize these compounds in greater detail, whole *N. benthamiana* leaves were extracted using two protocols to extract total and surface associated hydrophobic metabolites, respectively. Using total methanolic extracts of homogenized *N. benthamiana* leaves, compounds **11**, **12,** and **13** could all be identified as minor components (Figure 3). In contrast, **11**, **12,** and **13** were major metabolites when leaves were surface-washed with solvent, suggesting these metabolites selectively accumulated on the leaf surface. To confirm their identity, surface washes from multiple leaves were combined and the phenolics present concentrated and characterized (Table 1). All the compounds were identified as polymethylated flavonols, namely kaempferol-3,7-dimethylether (**11**), quercetin-3,7,3′,4′-tetramethylether (**12**), and kaempferol-3,7,4′-trimethylether (**13**). The minor polar compound **9** was tentatively identified as the 4′-*O*-glucoside of **11** (Figure 4).

Expression of GSTs *in planta* should be the best way to promote interactions between GSTs and physiologically relevant ligands. However, the inevitable tissue disruption on extraction into aqueous buffer has the potential to generate artifacts arising from the interaction between a GST and a ligand usually found in a different compartment, or the generation of ligands formed through chemical- or enzyme-mediated oxidation during extraction. To examine the effects of oxidation during processing, *in vitro* ligand fishing experiments were performed where metabolites were initially extracted in solvent and then passed over the immobilized GST, thereby reducing the potential for spontaneous, or enzyme-catalyzed oxidative reactions. In the case of the *At*GSTF2 ligands, while the flavonols and oxylipin conjugates observed in plant expression studies were consistently recovered, the flavonolignans were not identified, suggesting these were artifacts of the extraction protocol and not true metabolites.

### Identification of Ligands of GSTs Stably Expressed in Arabidopsis

Arabidopsis was stably transformed with constructs allowing the constitutive expression of *Strep*-tagged *At*GSTF2 and *At*GSTU19. The foliage of 12 week old plants was then harvested and the *Strep*-tagged GSTs isolated. Compared to the studies *in N. benthamiana*, *At*GSTF2 was only weakly expressed in Arabidopsis (Figure 2), with the associated poor recoveries of protein resulting in a failure to identify associated ligands. In contrast, *Strep*-tagged *At*GSTU19 was obtained in reasonable yield and found to co-purify with five UV-absorbing peaks that were not present in the controls (Figure 3C). These compounds (**14**, **15**, **16**, **18**, and **19**), were a series of related metabolites that eluted as a series of peaks, each differing in *m/z* value by +31.97 when analyzed by MS. Taking into account the increasing contribution of the M+2 isotopic ion, it was clear that the chemical composition of these compounds differed solely in the number of sulfur atoms present. UV spectral and MS-MS analysis suggested the presence of an indole moiety, while fragmentation with neutral losses of 75 Da and 129 Da were characteristic of glutathionylated conjugates (Supplementary Figure S1). The simplest compound in the series (**14**, Table 1), was identified as 3-methylindolyl glutathionyl disulfide (ISSG; Figure 4), with its identity confirmed after synthesizing a reference standard (Supplementary Figure S3). By analogy, the later-eluting compounds were the respective tri- (**15**), tetra- (**16**), penta- (**18**), and hexa-sulfide (**19**) analogs (ISS_n_G; Table 1 and Figure 4). The presence of disulfide bonds was confirmed by reducing the mixture of ligands (compounds **14–19**) with 40 mM DTT for 3 h at 4°C. This treatment released a single indole compound, identified as 3-thiomethylindole. To determine whether or not these indole conjugates were formed in the course of extraction, solvent extracts from Arabidopsis foliage were incubated with recombinant *At*GSTU19. ISSG together with low levels of ISSSG were identified, while the higher polysulfides were absent. In their place, 3-glutathionyl-*S*-methylindole (ISG; MH^+^ = *m/z* 437.15) was observed. This result suggested that the higher molecular weight disulfides were probably formed as a result of oxidative reactions during processing. Further non-UV absorbing ligands of *At*GSTU19 were identified as the glutathione conjugate of 12-oxo-phytodienoic acid (OPDA) **8**, and a related metabolite **17** with an elemental composition of GSH + C_18_H_30_O_3_. After subtraction of the glutathionyl-moiety, compound **17** was most likely a conjugate of the oxylipin keto-octadecadienoic acid. In the case of these conjugates, it was likely that their binding was a consequence of the catalytic activity of the ligandin, with *At*GSTU19 previously shown to catalyze the glutathionylation of OPDA (Dixon and Edwards, 2009).

### Binding and Inhibition of GSTs by *in vivo* Ligands

To investigate the binding characteristics of the ligands to the GSTs, isothermal titration calorimetry (ITC) was used to determine binding affinity and stoichiometry. With *At*GSTF2 the commercially available compounds kaempferol and kaempferol-3,7′-dimethylether were used, along with the methylated flavonols purified from the surface of the tobacco leaves (Table 2 and Supplementary Figure S4). Kaempferol was a relatively poor ligand (*K_d_* = 12 μM), with tighter binding determined with kaempferol-3,7′-dimethylether (**11**; *K_d_* = 0.22 μM) and kaempferol-3,7,4′-trimethylether (**13**; *K_d_* < 1 μM). The flavonols bound with a stoichiometry close to 0.5:1, suggesting a single high-affinity binding site per *At*GSTF2 dimer. When *At*GSTU19 was tested with ISSG, tight and stoichiometric binding was determined (Table 2 and Supplementary Figure S5). *At*GSTU19 enzyme activity, as determined by the GSH conjugation of 1-chloro-2,4-dinitrobenzene, was inhibited by ISSG in a competitive manner with respect to GSH, confirming that ISSG binding occurred at the enzyme active site.

**Table 2:** ITC-derived thermodynamic parameters for titration of the compounds listed into GSTF2 in the presence of 1 mM glutathione.

	Parameter		
Interaction (+cofactors)	N	K_a_ (μM^-1^)	ΔH (kcal/mol)
GSTF2 + kaempferol (+GSH)	0.60 ± 0.05	0.08 ± 0.01	-12.6 ± 1.4
GSTF2 + **13** (+ GSH)	0.54 ± 0.01	4.50 ± 0.29	-18.4 ± 0.2
GSTU19 + ISSG	0.98 ± 0.01	8.54 ± 0.76	-10.6 ± 0.1
GSTU19 + *S*-hexylglutathione	0.84 ± 0.01	3.22 ± 0.19	-11.8 ± 0.1

*In each case, parameters were obtained from fits to a one set of sites binding model, with errors representing standard error in the fitted model. Certain experiments contained GSH (1 mM)*.

## Discussion

Identifying compounds that selectively bind to the active and regulatory sites of proteins is a well established method in defining the substrates and catalytic intermediates of enzymes, and ligands for receptors (Shinohara and Matsubayashi, 2007). In addition, assaying for potent binding activity is a powerful route to discover small molecules that disrupt protein function through direct inhibition, or allosteric effects. Such inhibitors can then be used in applications in biomedicine and crop protection, with plant GSTs being an attractive target for selective chemical intervention given their multiple roles in stress tolerance of biotechnological interest (Nianiou-Obeidat et al., 2017). Classically, natural product ligands are discovered by fractionating crude biological extracts, screening preparations for biological activities against protein or cellular targets and then purifying and characterizing the compounds of interest (Weller, 2012). While such approaches have good provenance, the associated methods are time consuming and require the compounds to be isolated as being stable and present in quantities amenable for compositional and structural determination. As such, there is a real need to develop methods which accelerate the discovery of novel ligands, including intermediates which may be relatively unstable, and/or in low abundance as leads for new chemical intervention tools.

Using *Strep*-tagged GSTs as a test-bed, the current studies have demonstrated that previously undescribed ligands can be isolated from gram quantities of tissue following transient, or stable, expression in plants. The retained ligands could then be identified in yields allowing them to be identified by high resolution MS. Such ‘ligand-fishing,’ is a potentially powerful technology to both identify natural product high affinity binding partners of proteins as well as shedding light on their potential endogenous functions *in vivo*. The power of the approach was that it allowed for the recovery of protein-specific ligands that in several cases were unstable and therefore unlikely to be identified using conventional screening of total plant extracts. For both *At*GSTF2 and *At*GSTU19, the efficiency of ligand discovery was dependent on the level of protein expression *in planta*, the specificity and strength of recognition and the availability of low molecular weight binding partners in the host cell. The ligands identified could be functionally divided into those which were formed as artifacts of extraction, natural products recognized as reaction product orthologues and biologically active metabolites that were not linked to any known enzyme activity related to the GSTs.

With respect to artifacts, a comparison of the ligands bound when *At*GSTF2 was recovered from being expressed transiently in *Nicotiana* with those retained by the enzyme when solvent extracts from the identical plant tissue were passed over the immobilized protein, strongly suggested that the glutathionylated lignanamides (**5, 6, 7**) were artifacts formed during extraction. While the lignanamides cannabisin, grossamide and grossamide K have been described previously in plants (Yoshihara et al., 1981; Sakakibara et al., 1992; Seca et al., 2001), this is the first report of the corresponding glutathione conjugates being determined. The conjugates appear to be generated when feruloyltyramines are mixed with GSH in the presence of a radical-generating enzyme such as horseradish peroxidase acting on hydrogen peroxide. A similar mechanism has been described for ascorbate peroxidases catalyzing the glutathionylation of phenylpropanoids (Dean and Devarenne, 1997). Intriguingly, *At*GSTU19 did not bind to these ligands even though it did bind to a glutathionylated conjugate of the phenylpropanoid derivative chlorogenic acid (**3**). This may suggest that either the two GSTs have fundamentally different binding affinities for the liganamide conjugates, or that during extraction, *At*GSTF2 was transiently in closer proximity to the generation of these artifacts than *At*GSTU19. The functional significance of the binding to these ligands is unknown, though there is one report of feruloyltyramine weakly inhibiting GSTUs from poppy (Yu and Facchini, 2000). In the case of *At*GSTU19, evidence was obtained that at least some of the higher order polysulfides of glutathionylated 3-methylindole (**14**) isolated following expression in Arabidopsis were also formed as extraction artifacts. These derivatives have not been described previously and are presumably derived from indole-3-carbinol, a known degradation product of antifungal glucosinolates. The indole-3-carbinol is then proposed to interconvert to a reactive 3-methyleneindolenine intermediate that can react spontaneously with GSH to form a thiolated product (Staub et al., 2002). Our current work suggests that this reaction occurs in Arabidopsis and that the glutathione conjugate is enzymically processed to 3-thiomethylindole by a C-S lyase. The 3-thiomethylindole then forms a mixed disulfide with GSH to give **14**, with higher order di- tri- and tetra-sulfides forming through radical-enhanced disproportionation during the course of extraction. Thus, while the presence of 3-thiomethylindole would be likely to be a true product resulting from processing an endogenously formed glutathionylated detoxification product, the observed polysulfides would be most likely maintained at very low levels *in planta*, or only produced under conditions of oxidative stress.

The next group of ligands to be identified were glutathionylated natural products that represented examples of intermediates of GST-mediated metabolism *in planta*. Of these the glutathione conjugates of fatty acid oxylipin derivatives (**8, 10, 17**), detected bound to *At*GSTF2 in *N. benthamiana* and *At*GSTU19 in Arabidopsis were good examples. There has been significant interest in the *S*-glutathionylation of 12-oxo-phytodienoic acid (OPDA) and related compounds, with the conjugates accumulating in plants fed with oxylipins or infected with bacterial pathogens (Davoine et al., 2005, 2006). The lack of observed conjugate binding to *At*GSTF2 in arabidopsis and to *At*GSTU19 in *N. benthamiana* was most likely due to assay sensitivity and the relative levels of expression of the two *Strep*-tagged proteins in these plant hosts. Another key difference may be that while *At*GSTU19 could catalyze the formation of the oxylipin-glutathione conjugate, *At*GSTF2 could only capture the conjugate following its formation elsewhere. It is of interest that *At*GSTU19 catalyzes the formation of the OPDA conjugate, an activity previously reported for *At*GSTF8 in arabidopsis (Mueller et al., 2008). To date the physiological consequence of these conjugation reactions in plants are unknown, though similar derivatives of the chemically related leukotrienes are known to control the biological activity of chemically related prostraglandins in mammals (Wang and Ballatori, 1998).

Finally there were those ligands representing compounds that had no functional link to the catalytic activities of GSTs, but point to important endogenous functions in the binding and transport of secondary metabolites. The ligand fishing studies with *At*GSTF2 in *N. benthamiana* identified a series of polyphenolic metabolites, identified as methylated derivatives of kaempferol and quercetin. In their unmethylated forms these flavonols had previously been shown to bind weakly to *At*GSTF2 (Smith et al., 2003), along with synthetic aromatics such as naphthalene acetic acid and 1-*N*-naphthylphthalamic acid (Zettl et al., 1994; Smith et al., 2003). The significance of flavonoid binding by plant GSTs has been most clearly demonstrated in genetic studies with the tau class AN9 GST from petunia and the phi GSTs Bz2 in maize and TT19 (=*At*GSTF12) in arabidopsis (Marrs et al., 1995; Mueller et al., 2000; Kitamura et al., 2004). Unlike *At*GSTF12, *At*GSTF2 is not essential to anthocyanin pigment biosynthesis in arabidopsis (Dixon et al., 2005). However, *At*GSTF2 has been linked to flavonoid metabolism *in planta*, showing reduced levels of expression in *tt4* flavonoid-deficient mutants and a relocation to the cytosol rather than the plasma membrane (Smith et al., 2003). Recent structural biology studies have revealed a specific binding pocket for flavonoids in *At*GSTF2 remote from the active site at the dimer interface of the protein (Ahmad et al., 2017). The current binding studies confirm that AtGSTF2 dimers bind a single molecule of kaempferol, or related methylated derivatives (Table 2), further pointing to a very specific interaction of this protein with flavonoids. In *N. benthamiana*, *At*GSTF2 selectively bound the hydrophobic flavonoids associated with the leaf surface, consistent with an association of the protein with membranes and extracellular deposition of hydrophobic natural products. In arabidopsis, no flavonoids were associated with *At*GSTF2, but both crude and surface-extracted metabolites in arabidopsis lacked the hydrophobic flavonoids seen in *N. benthamiana*.

Collectively, the current ligand fishing studies have identified a group of GST binding partners not previously identified in experiments conducted in recombinant bacteria. Our results demonstrate that the different GST classes exhibit a degree of ligand specificity. Predictably, ligands such as flavonoids derived from species-specific secondary metabolism clearly differed in the ligand fishing experiments in bacteria and plants. In the case of the more conserved primary metabolism the GSTUs were found to bind glutathionylated fatty acid derivatives in both plants and *E. coli*. In contrast the flavin and porphyrin metabolites that are core primary metabolites in the different hosts were only determined in the experiments in *E. coli*. *At*GSTU19 was only one of two Arabidopsis GSTs that selectively bound the glutathione conjugates of the heme precursors harderoporphyrin (ogen) (**1**) and protoporphyrin (ogen) (**2**) when expressed in *E. coli*, but failed to retain these ligands when present in either plant hosts. Based on previous studies with porphyrin binding maize GSTUs, this failure to bind these metabolites *in planta* is most likely because GSTU19 is predominantly expressed in the cytosol whereas these metabolites are synthesized in the chloroplast (Dixon et al., 2008).

Our studies shed further light on the ability of plant GSTs to bind, and in some cases glutathione-conjugate natural and synthetic ligands, which may well influence the availability, and biological activity of such ligands. While the ligands identified here are diverse, they represent many chemistries commonly encountered in plants, with the selectivity in recognition shown in just *At*GSTF2 and *At*GSTU19 demonstrating how these compounds could perform multiple and species-specific roles relating to signaling, transport and storage roles in both primary (fatty acid) and secondary (phenolic, alkaloid, and glucosinolate) metabolism, as has been recently proposed (Labrou et al., 2015). Using the results of these ligand capture experiments it will now be of interest to carry out directed metabolomic experiments to see if disruption of the expression of these ‘ligandin’ GSTs causes subtle alterations in these respective branches of metabolism under differing conditions of stress and plant growth.

## Author Contributions

DD and RE conceived the experiments, interpreted the data and wrote the manuscript. DD conducted the experimental work.

## Conflict of Interest Statement

The authors declare that the research was conducted in the absence of any commercial or financial relationships that could be construed as a potential conflict of interest.

## Footnotes

1 www.cambia.org
