# Supplementary material for: Protein-Ligand Fishing in planta for Biologically Active Natural Products Using Glutathione Transferases
Source: Front Plant Sci. 2018 Nov 15;9:1659. doi: 10.3389/fpls.2018.01659 (PMC6253249; doi:10.3389/fpls.2018.01659)
Supplement: Supplementary file 1 [file Data_Sheet_1.pdf]

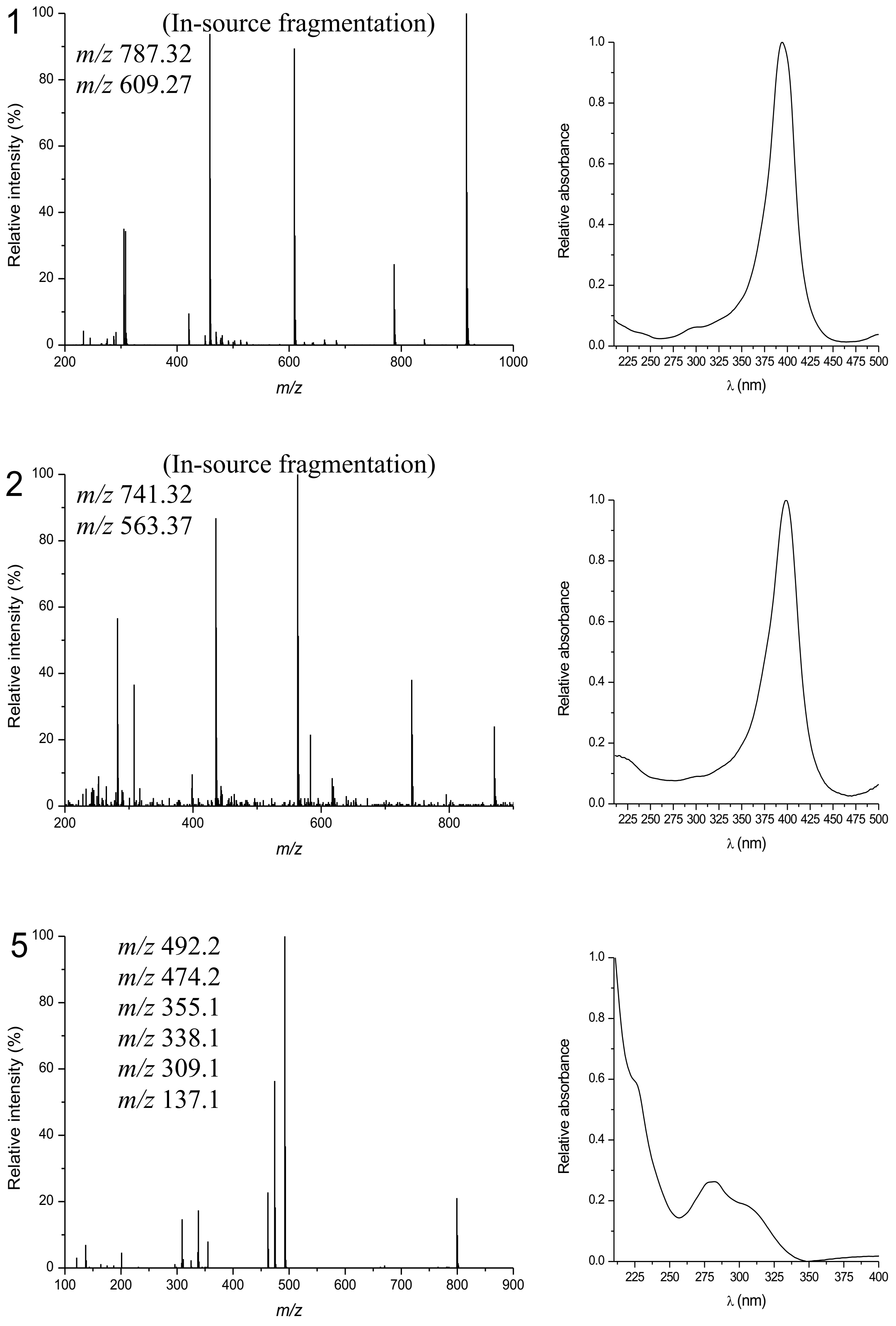

Supplementary Figure S1 (1 of 5). CID mass spectra and UV spectra for GST ligands, with major mass ions as listed.

Supplementary Figure S1 (2 of 5)

6

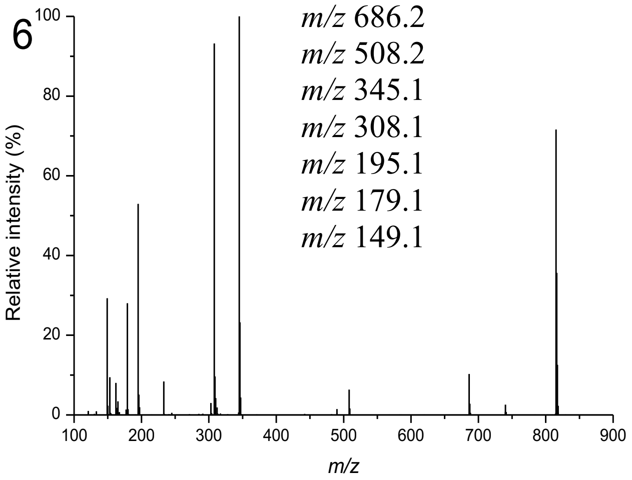

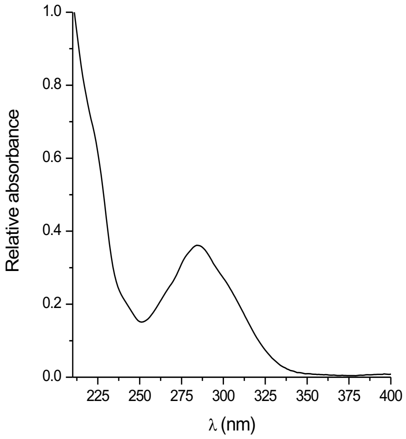

7

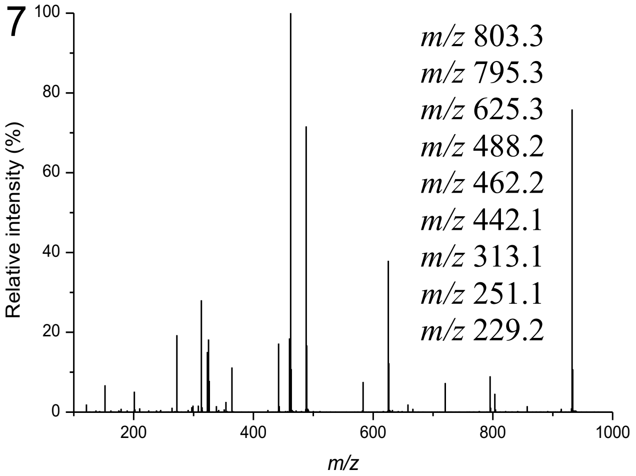

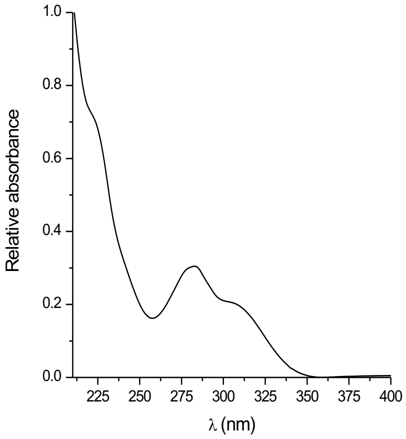

9

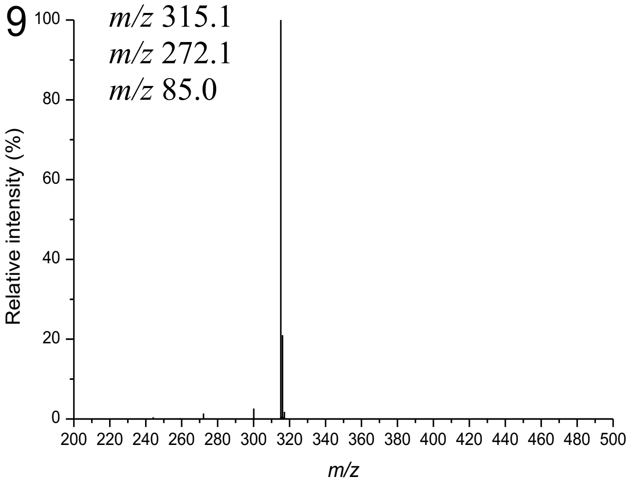

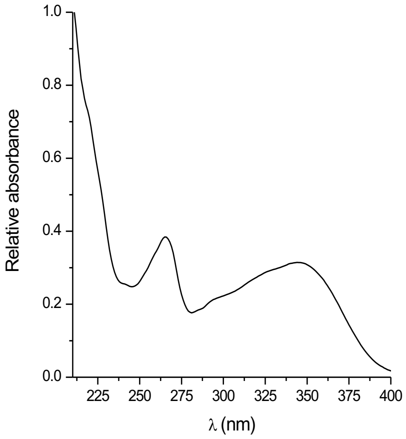

Supplementary Figure S1 (3 of 5)

10

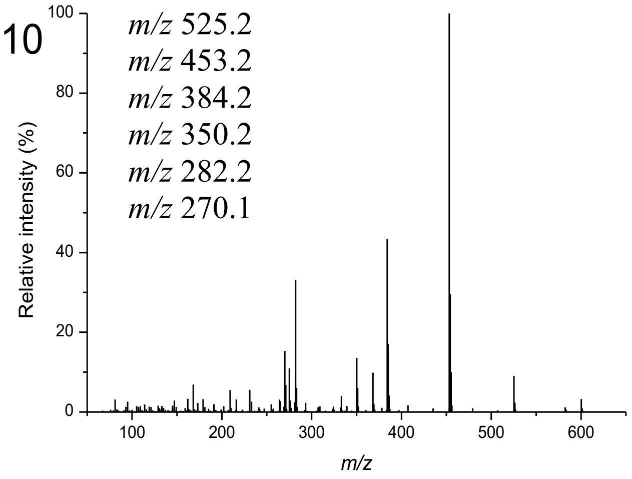

11

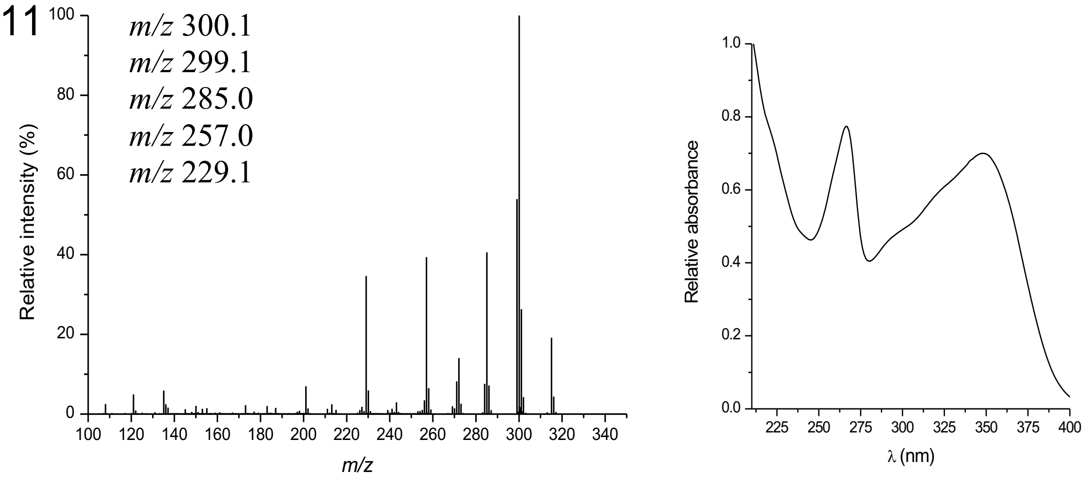

12

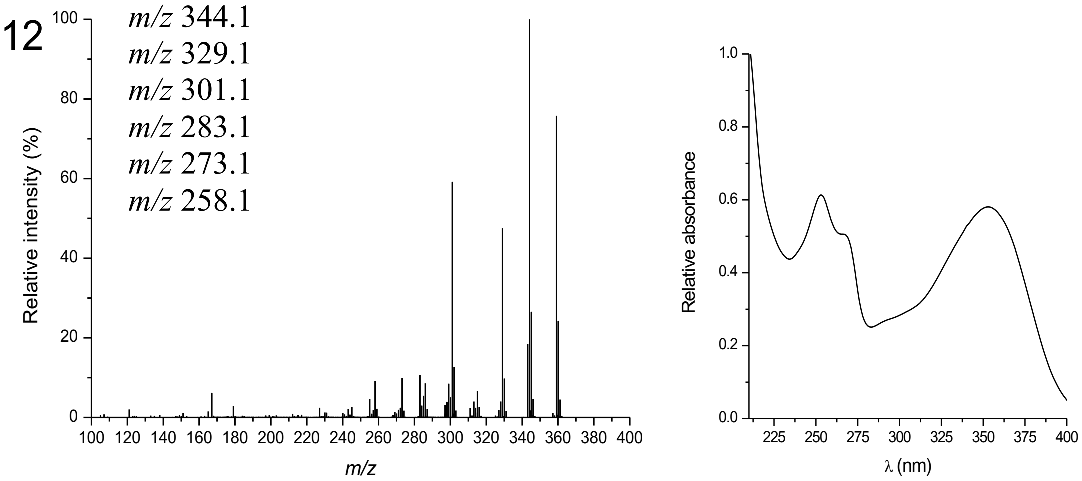

Supplementary Figure S1 (4 of 5)

13

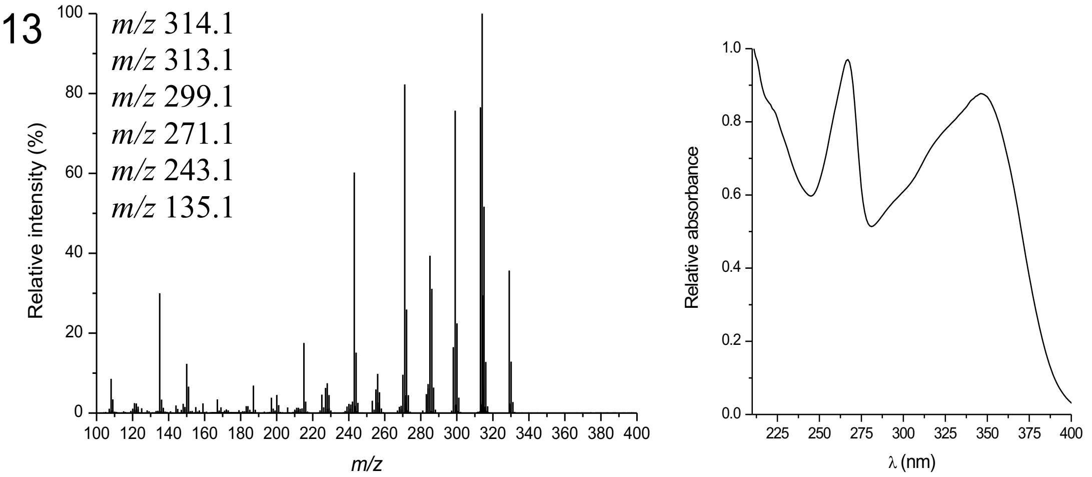

14

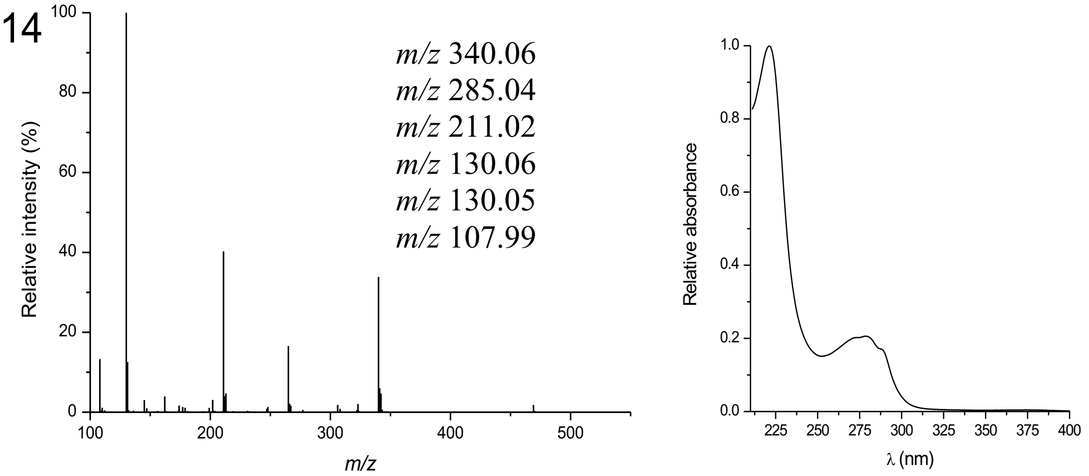

15

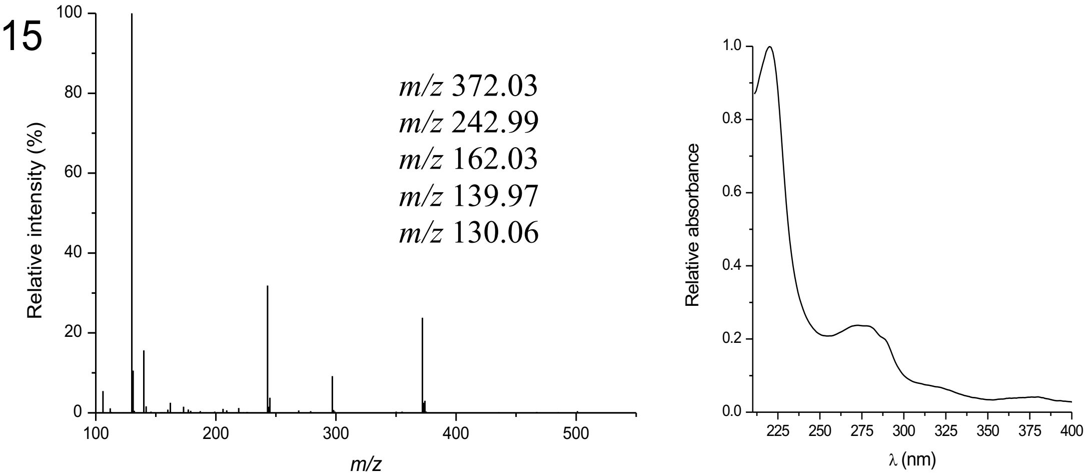

Supplementary Figure S1 (5 of 5)

16

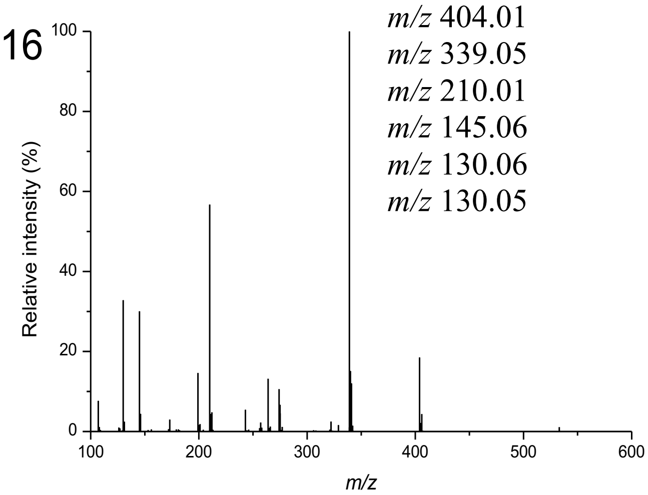

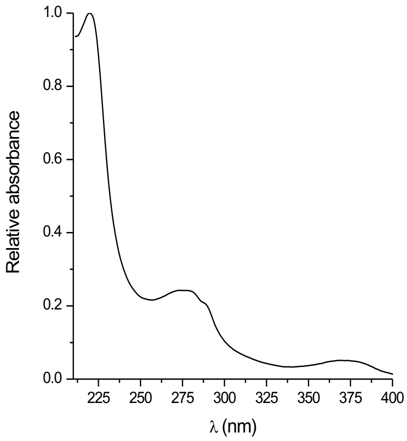

17

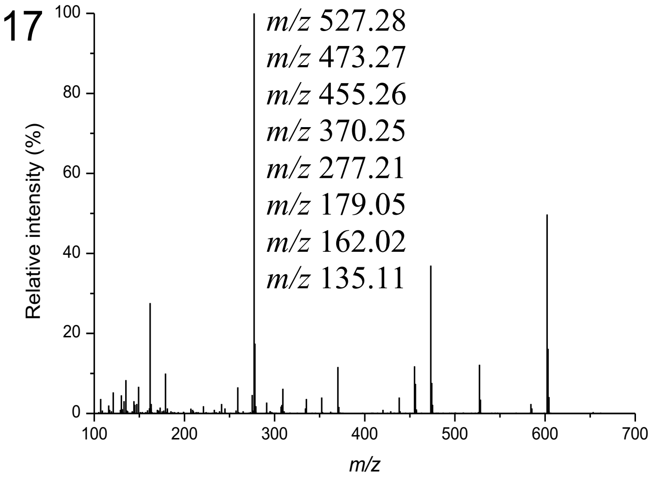

18

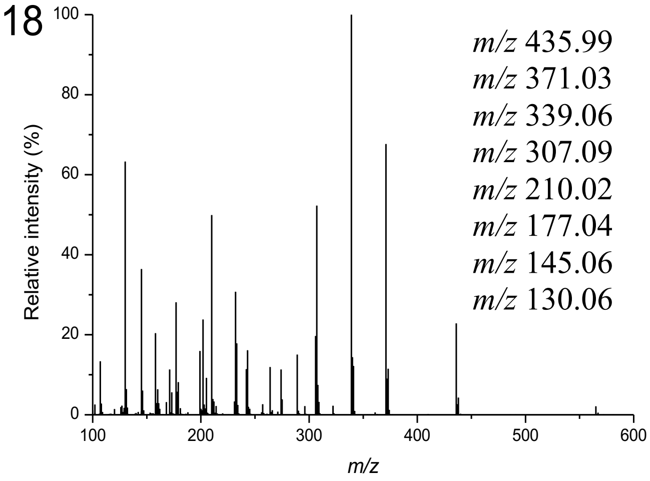

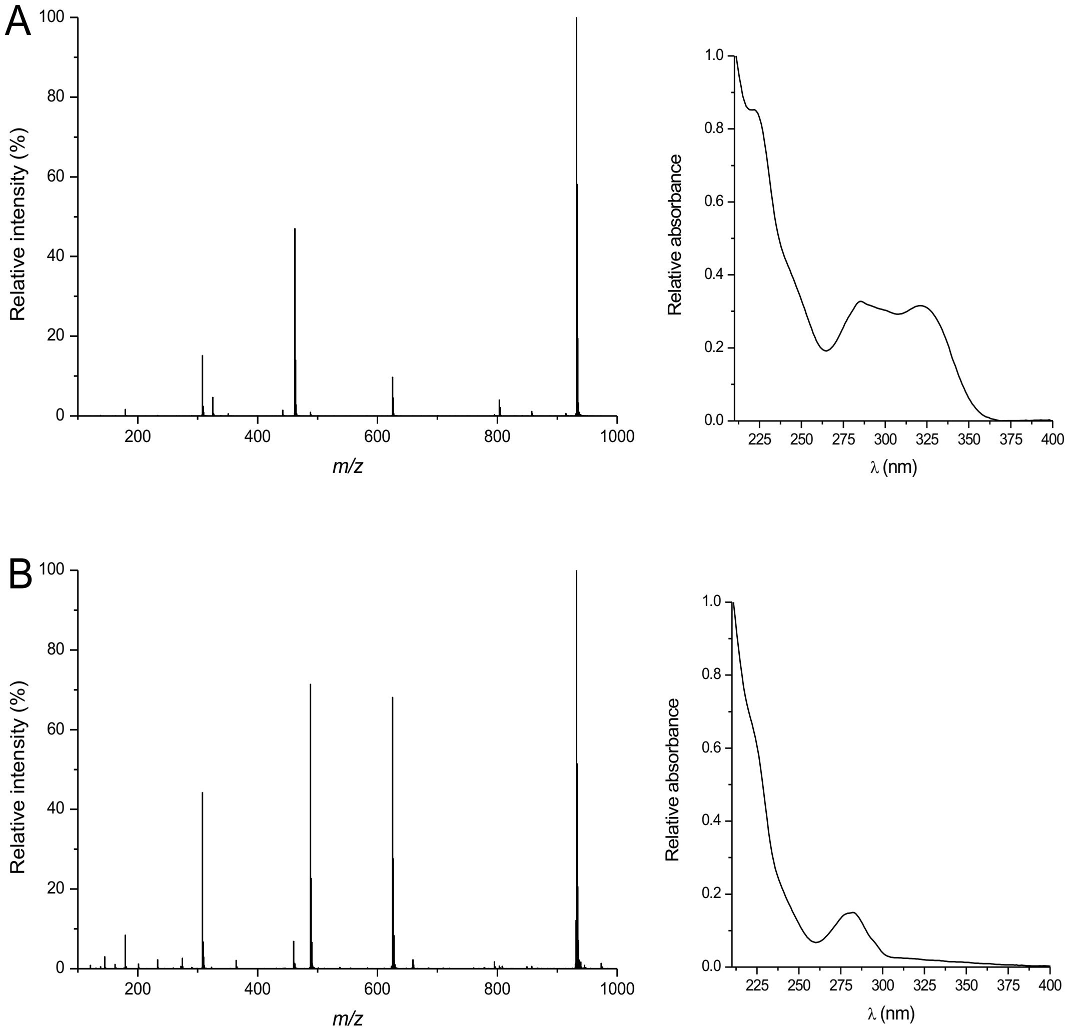

Supplementary Figure S2. CID mass spectra and UV spectra for chemically synthesised compounds related to compound **7**. A. Feruloyltyramine dimer synthesised by $FeCl_3$ treatment, then glutathionylated by addition of horseradish peroxidase, GSH and H_2O_2. B. Feruloyltyramine oxidatively glutathionylated by addition of horseradish peroxidase, GSH and H_2O_2. Neither standard was an exact match to **7**, but both had similar properties suggesting that the 2 standards and compound **7** were all structural isomers.

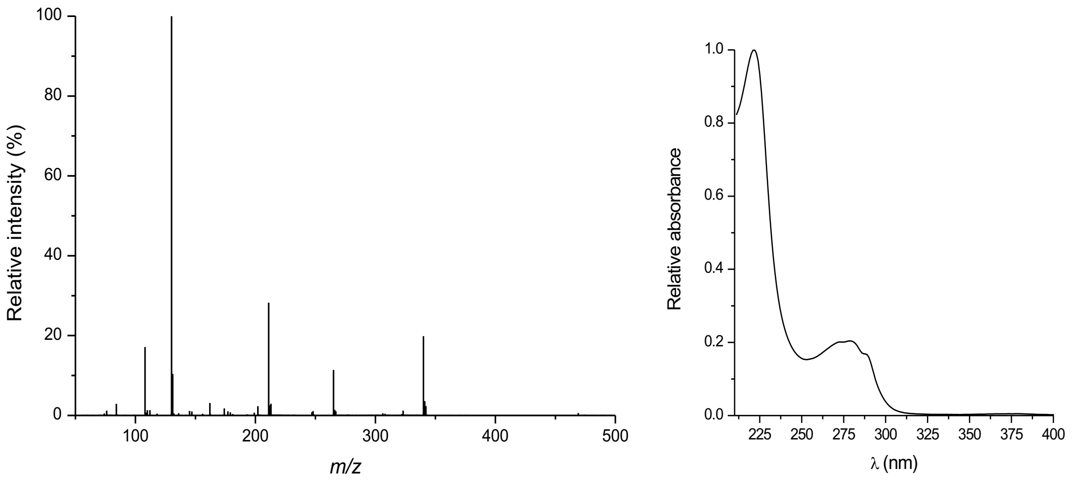

Supplementary Figure S3. CID mass spectrum and UV spectrum for chemically synthesised standard matching compound **14**, 3-methylindolyl glutathionyl disulfide (ISSG).

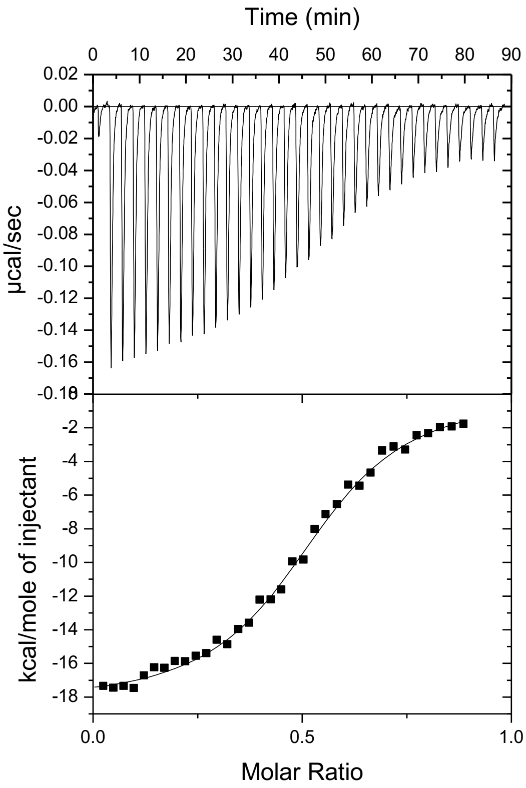

Supplementary Figure S4. Typical ITC data for GSTF2 (7.7 μM) binding to compound **11** (putative kaempferol-3,7'-dimethylether). The experiment was performed in HBS containing 1 mM GSH, 0.1% Tween 20 and 1% ethanol. The associated line indicates best fit curves for a one set of sites binding model.

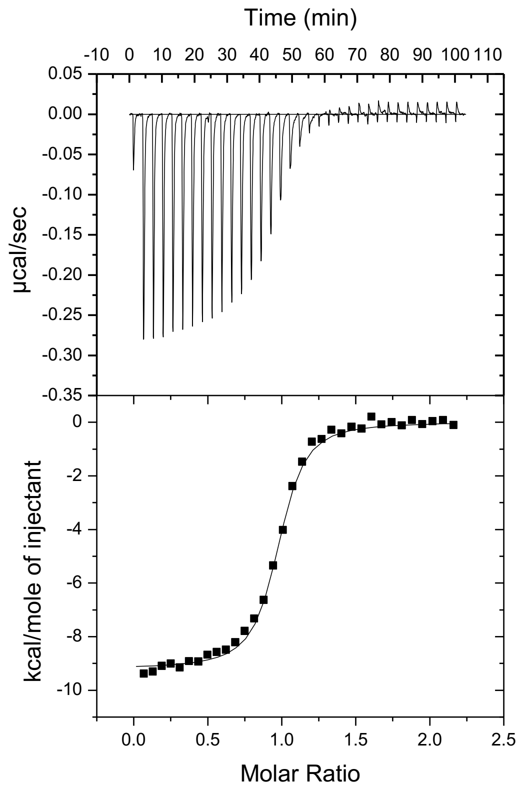

Supplementary Figure S5. Analysis of GSTU19 ligand binding by ITC. Binding of ISSG to 11.1 uM GSTU19. A best fit line corresponding to a one set of sites binding model is shown.
